# You Are What You (First) Eat

**DOI:** 10.3389/fnhum.2018.00323

**Published:** 2018-08-13

**Authors:** Kelly L. Buchanan, Diego V. Bohórquez

**Affiliations:** ^1^School of Medicine, Duke University, Durham, NC, United States; ^2^Department of Medicine, Duke University, Durham, NC, United States; ^3^Department of Neurobiology, Duke University, Durham, NC, United States

**Keywords:** enteroendocrine cell, gut-brain axis, sensory neurodevelopment, fetal microbiome, prenatal nutrition

## Abstract

As far back as we can remember, we eat. In fact, we eat *before* we can remember. Our first meal is amniotic fluid. We swallow it during the first trimester of gestation, and with that, we expose our gut to a universe of molecules. These early molecules have a profound influence on gut and brain function. For example, the taste of the amniotic fluid changes based on the mother’s diet. Indeed, recent findings suggest that food preferences begin *in utero*. Likewise, a baby’s first exposure to bacteria, previously thought to be during birth, appears to be *in utero* as well. And just as postnatal food and microbiota are implicated in brain function and dysfunction, prenatal nutrients and microbes may have a long-lasting impact on the development of the gut-brain neural circuits processing food, especially considering their plasticity during this vulnerable period. Here, we use current literature to put forward concepts needed to understand how the gut first meets the brain, and how this encounter may help us remember food.

## Introduction

Before we are born, we must learn to hear, smell, taste—and eat. The first meal a human consumes is not its mother’s milk or formula, but instead amniotic fluid ingested by the fetus *in utero*. That first meal introduces the brain and the gut, establishing communication which will be sustained throughout its lifespan.

During gestation, the fetus recognizes the scent of its amniotic fluid (Marlier et al., 1998; Schaal et al., 1998), and even its taste (Lipchock et al., 2011). After the first ingestion, this fluid has a profound effect on the morphological development of the gastrointestinal tract (Mulvihill et al., 1985; Bohórquez et al., 2011). How this first meal influences our ability to sense food remains unexplored. Only in recent years have the means through which the gut senses chemical components of food become evident (Jang et al., 2007).

**Figure 1:**
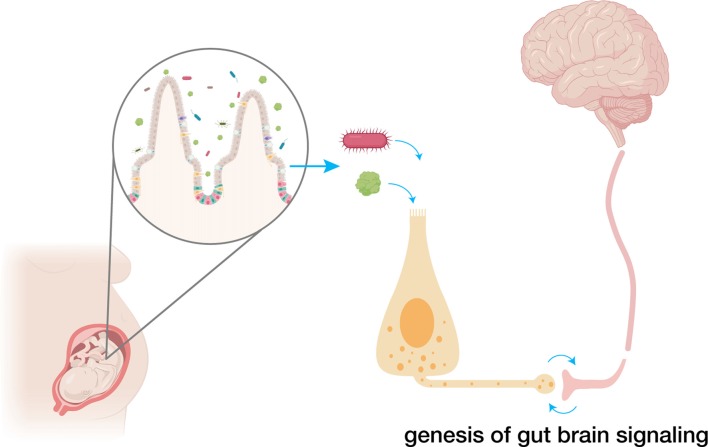
Early exposure to amniotic fluid, nutrients and the microbiota influences gut-brain circuit development. Prior to birth, fetal swallowing exposes its gut to amniotic fluid, containing both nutrients and a developing microbiome. There, nutrients, microbiota and their metabolites come in contact with developing gut sensor cells—enteroendocrine cells—which form synapses with vagal nodose neurons. Dysregulation of the development of this gut-brain neural circuitry can have profound, prolonged effects on later health. Created with BioRender.

Soon after the discovery of taste receptors in the tongue (Adler et al., 2000), it became clear that those receptors were not confined to the tongue, but are also prominently expressed throughout the gastrointestinal epithelium (Rozengurt and Sternini, 2007). Those taste receptors are located in specialized intestinal sensory epithelial cells, including enteroendocrine cells. And just like other sensory epithelial cells, enteroendocrine cells are innervated (Bohórquez et al., 2015). This synapse is the first link, the first pathway, from the gut to the brain. It serves as a sensory portal for the transduction of stimuli from the gut lumen, and as a path for pathogens to access the peripheral and central nervous systems. We draw information from other sensory systems to infer potential implications of early sensory stimuli on the development and function of gut-brain sensory transduction (Figure 1). These lines, rather than being an exhaustive account, should serve as a foundation to stimulate the development of knowledge of the gut-brain sensory transduction field.

## The First Enteroendocrine Cell

*What is known about enteroendocrine cell developmental origin can be traced to the* *1960s*. Anthony Pearse discovered that seemingly unrelated endocrine cells in different organs appeared to have a common origin—the neural crest (Pearse, 1969). This grouping of cells, known as amine precursor uptake and decarboxylation (APUD) cells, has been heavily debated (Le Douarin and Teillet, 1973; Andrew et al., 1998; Barker and Clevers, 2007; Barker et al., 2008) and eventually fell out of favor. Nonetheless, like other sensory epithelial cells of endodermal origin, enteroendocrine cells have prominent neuronal features. They are electrically excitable (Rogers et al., 2011), form synapses (Bohórquez et al., 2015), and release neuropeptides and neurotransmitters in response to stimuli (Hartenstein et al., 2017). Because of the mounting evidence that these cells act as true epithelial transducers, further exploration will elucidate the precise origin of these cells.

The first enteroendocrine cells appear early in gestation, during gut tube development. At this point the cells remain mitotically active (Crosnier, 2005; Penkova et al., 2010; Hartenstein et al., 2017). Upon folding of the gut epithelium into crypts and villi, most cells are differentiated, and further proliferation occurs from the Lgr5-expressing adult stem cells of the crypts (Barker et al., 2007). The differentiation of an intestinal stem cell into an enteroendocrine cell is dependent on Delta-Notch signaling (Micchelli and Perrimon, 2006). Low levels of Notch influence enteroendocrine differentiation, while high levels of Notch promote an enterocyte fate. Then, in the presence of low Notch activity, enteroendocrine cell fate is driven by acheate-scute (ac-sc) complex genes that encode bHLH transcription factors and the enteroendocrine cell fate promoter Prospero, among others (Yin and Xi, 2018).

The role of ac-sc complex gene expression in driving neuronal differentiation was established in *Drosophila* decades ago (Cubas et al., 1991; Skeath and Carroll, 1991). Since then, ac-sc complex gene expression has been found to promote enteroendocrine cell fate (Bardin et al., 2010). Just recently, Chen and colleagues used a *Drosophila* model to propose that Delta-Notch signaling guides enterocyte generation from intestinal stem cells as the default mode, and that, paralleling neuronal differentiation, transient Sc expression is required to trigger enteroendocrine cell formation (Chen et al., 2018). In mammals, the bHLH transcription factor family is commonly known to be pro-neural, but certain bHLH transcription factors including Math1 (Atoh1), Neurogenin3, and NeuroD have also been identified as pro-endocrine. Math1 is expressed in cells destined to become secretory cells (Yang et al., 2001). Then, neurogenin3 expression is required for intestinal enteroendocrine cell development (Jenny et al., 2002; Schonhoff et al., 2004). NeuroD controls terminal differentiation of specific enteroendocrine cells (Naya et al., 1997; Mutoh et al., 1998). In parallel or downstream to ac-sc and bHLH signaling, Prospero functions to commit intestinal stem cell differentiation to enteroendocrine cells (Zeng and Hou, 2015). Though the discovery of its function in enteroendocrine cells is recent, Prospero has long been studied as a transcription factor predominantly found in the nervous system (Manning and Doe, 1999).

At a more macroscopic level, the similarities between the differentiation of enteroendocrine cells and neural cells persist. It has been suggested that enteroendocrine cells can be generated from intestinal stem cells using one of three pathways: terminal differentiation into an enteroendocrine cell, asymmetric division into an intestinal stem cell and an enteroendocrine cell, or symmetric division into two enteroendocrine cells (Zeng and Hou, 2015). Experiments from Chen and colleagues in *Drosophila* suggest that intestinal stem cells actually yield enteroendocrine progenitor cells, which then terminally divide into two enteroendocrine cells (Chen et al., 2018). This proposed process of cell division mirrors ganglion mother cells in *Drosophila* neuroblasts: each neuroblast divides exactly once prior to terminal differentiation into neurons or glial cells (Homem and Knoblich, 2012). Regardless of method of division, intestinal stem cells are located basally; similarly, sensory organ progenitors of *Drosophila* that give rise to sensory neurons are located sub-epidermally. And in both, polarity is acquired after differentiation (Hartenstein et al., 2017). Thus, when Notch activity is low, ac-sc complex genes, bHLH transcription factors and Prospero signaling drive enteroendocrine cell development from intestinal stem cells by inducing the formation of an enteroendocrine progenitor cell that divides into enteroendocrine cells.

## When the Gut First Meets the Brain

The discovery that enteroendocrine cells are innervated opened a path for gut sensory transduction. Such signals could travel from the gut lumen to the brain, via the vagus nerve. The vagus is the primary integrator of visceral sensory information, and vagal innervation of the gastrointestinal tract is critical to various homeostatic processes including satiety and visceral pain signaling (Berthoud, 2008; Ratcliffe et al., 2011b). Additionally, the vagus has been shown to be critical in the communication of the microbiome with the central nervous system. Chronic exposure to *Lactobaccillus rhamnosus* has anxiolytic effects in mice that are eliminated by subdiaphragmatic vagotomy (Bravo et al., 2011).

Vagal innervation of the gastrointestinal tract begins early in embryonic life. In one study of fetal mice, vagal sensory fibers reached the stomach by embryonic day 12, the duodenum by day 14, and the distal small intestine by day 16 (Ratcliffe et al., 2011a). By visualizing vagal fibers with DiI applied to the fetal nodose ganglia, vagal fibers appear to be attracted to Netrin-1 expressed by the developing foregut through deleted in colorectal cancer receptors (Ratcliffe et al., 2006). Attractive signaling through neurotrophins including brain-derived neurotrophic factor (BDNF) and repulsive signaling through Slit/Robo are also critical to the prenatal development of sensory vagal innervation of the gastrointestinal tract.

BDNF is crucial for synapse regulation (Lu et al., 2014). Centrally, hippocampal BDNF expression and neurogenesis is dependent on the vagus nerve (O’Leary et al., 2018). Peripherally, neurotrophin-3, neurotropin-4 and BDNF promote vagal innervation of the gastrointestinal tract. In knockout mice of neutrotropin-3 or neurotrophin-4, severely reduced vagal innervation is observed in the esophagus (Raab et al., 2003) and small intestine (Fox et al., 2001), respectively. In a homozygous BDNF knockout, postnatal day 0 mice exhibit altered morphology and reduced density of stomach vagal afferent structures such as intramuscular arrays and intraganglionic laminar endings compared to wild-type controls (Murphy and Fox, 2010). BDNF is known to be expressed in the muscle layers of the GI tract (Murphy and Fox, 2010), and indeed, limited studies have suggested that enteroendocrine cells of higher vertebrates express neurotrophic factors including BDNF and neurotropin-3 (Lucini et al., 2002). *In vitro*, when a murine enteroendocrine cell is co-cultured with a sensory neuron there is a clear affinity. The neuroepithelial circuit is recapitulated; enteroendocrine cells synapse with vagal nodose neurons *in vitro*, and time-lapse footage suggests that enteroendocrine cells guide the process (Bohórquez et al., 2015).

Repulsive signaling also works to counter attractive signaling and restrict vagal afferents from entering the bowel wall; Slit/Robo signaling is the most well-studied pathway. Transcripts encoding Robo1-2 have been identified in the embryonic and adult nodose ganglia; Slits1–3 in the embryonic and adult gut (Goldberg et al., 2013). In the same study, co-cultures with mouse nodose ganglia and Slit2 secreting cells demonstrated chemorepulsion of nodose neurites by Slit2. Similarly, Slit/Robo signaling functions in enteroendocrine cell differentiation and development. In *Drosophila*, enteroendocrine cells release Slit1, which acts on Robo2 receptors in intestinal stem cells to limit differentiation to an endocrine fate, acting as a negative feedback control (Biteau and Jasper, 2014). Although the specific relationship of enteroendocrine-secreted Slit and vagal innervation of the gastrointestinal tract has not been established, juxtaposing these studies suggests a correlation. The repulsion-attraction mechanisms in enteroendocrine cells could serve as beacons for proper innervation of the intestinal mucosa.

Limited studies also suggest that vagal innervation of the gastrointestinal tract promotes survival and proliferation of enteroendocrine cells. In mice and calves, vagotomy reduces the density of enteroendocrine cells in the stomach (Qian et al., 1999; Soehartono et al., 2002). Although the moment of synapse formation between the vagus nerve and enteroendocrine cells remains unknown, the early development of vagal innervation and similarities in signaling molecules suggest that the gut and brain contact early in embryonic life. An early, synaptic, gut-brain connection could help us understand how we process early gut sensory stimulants—nutrients and bacteria.

## The Gut’s First Feeling: Nutrients and the Microbiome

The first drink of a mother’s milk is not a newborn’s first meal. Longitudinal, ultrasonographic studies of healthy, developing fetuses have demonstrated that as early as gestational week 12, newborn babies swallow amniotic fluid and by 18 weeks, gestational suckling begins (de Vries et al., 1985; Miller et al., 2003). Amniotic fluid contains the essential nutrients—glucose, fructose, fatty acids and amino acids—that the fetus will need to sense and absorb postnatally to survive. In fact, amniotic fluid provides a significant portion of nutrition to the fetus, and the inability to swallow is a risk factor for low birth weight and poor gastrointestinal development (Mulvihill et al., 1985; Bagci et al., 2016). However, the process of swallowing does more than allow the fetus to absorb the nutrients in amniotic fluid. Swallowing exposes the fetus’s orosensory and post-oral sensor cells—enteroendocrine cells—to nutrients and bacterial ligands that influence the development of gut-brain communication.

### Early Nutrient Sensing

The development of flavor memories begins *in utero* and influences later food preferences. In response to sweet solutions, ovine fetuses increase their rate of swallowing, demonstrating motivated behavior for rewarding substances (El-Haddad et al., 2002). In a well-designed, randomized, controlled trial, babies whose mothers drank carrot juice during the last trimester of gestation or the first 3 months of lactation were more accepting of carrots at 6 months of age (Mennella et al., 2001). In a subsequent study, formula fed infants were randomized to receive palatable cow milk-based formula or unpalatable protein hydrolysate formula at varying ages and durations. They found a sensitive period—prior to 4 months of age—when exposure to a flavor determines its hedonic tone (Mennella et al., 2011).

Both gustatory and post-oral signaling pathways contribute to the findings in these studies in fetuses and infants, but work in adult mice has shown that post-oral signaling, likely via enteroendocrine cells, can potently drive preference independent of taste. Intragastric sugar infusions can rapidly stimulate the intake and preference for non-nutritive solutions in mice (Sclafani and Ackroff, 2012). Furthermore, sweet blind *Trmp5* knockout mice can develop a preference for sucrose in a dopamine-dependent pathway based solely on its caloric value (de Araujo et al., 2008). Therefore, post-oral stimuli and their interactions with enteroendocrine cells are also likely to significantly influence the development and function of gut-brain neural circuits.

### The Fetal Microbiome

The amniotic fluid that constitutes a fetus’s first meal contains more than nutrients—it also carries microbiota. Recently, the dogma that the uterus is an immune-privileged environment has been challenged. If the fetus is exposed to microbes, studying intestinal sensation of nutrients in isolation would be only part of the puzzle. It is crucial to assess how a prenatal microbiome could affect gut-brain neural circuit development.

Microbiota play a critical role in the embryogenesis of many lower species. In lumbricid earthworms, marine sponges and caridean shrimp, for example, symbiotic bacteria are selectively recruited and vertically transmitted to developing embryos to support embryogenesis and protect them from pathogen colonization (Gil-Turnes et al., 1989; Ereskovsky et al., 2005; Davidson and Stahl, 2008). The same dependence on microbiota in higher species has not been established, but increasing evidence points toward vertical transmission of commensal prenatal microbes in higher vertebrates. In turkeys, microbial structures are clearly observed in the embryo’s intestinal tract well before hatching (Bohórquez, 2010). Jiménez and colleagues established the possibility of vertical transmission from mother to fetal amniotic fluid in mice (Jiménez et al., 2005). They isolated labeled *Enterococcus faecium* in the amniotic fluid of pregnant mice orally inoculated with the same strain; the bacterium was not isolated in control mice. In the same study, PCR analysis of umbilical cord blood of healthy mouse neonates delivered at term by cesarean section identified bacteria belonging to species including *Enterococcus faecium, Propionibacterium acnes, Staphylococcus epidermidis and Streptococcus sanguinis* (Jiménez et al., 2005). This was one of the first studies to suggest the presence of prenatal microbiota in healthy pregnancies.

Furthermore, in humans, studies have established distinct microbiota populations in the amniotic fluid, meconium, placenta and umbilical cord (Jiménez et al., 2008; Aagaard et al., 2014; Ardissone et al., 2014; Collado et al., 2016). In one study, over 5 in 10 human infants delivered at greater than 33 weeks of gestation had evidence of intestinal colonization by 16S rRNA amplification and sequencing of meconium samples (Ardissone et al., 2014). In this and corroborating studies, shared features and specific genera of microbiota between the meconium, amniotic fluid, and the placenta have been observed (Ardissone et al., 2014; Collado et al., 2016). The process of swallowing non-sterile amniotic fluid likely inoculates the healthy fetal gut with a developing microbiome.

### Early Influences of the Microbiome on Development and Disease

The birthing process further colonizes the newborn gut with microbial flora. Any disruption—including changes in birthing method and maternal perinatal antibiotics—can increase the risk of disease later in life. Using obesity as an example, in a study of 436 mother child-dyads followed for 7 years after birth, the risk for obesity was 84% greater in children whose mothers had taken late term antibiotics and 46% greater in children born by cesarean section (Mueller et al., 2015). In a subsequent study, the association between childhood obesity and cesarean section remained after controlling for maternal obesity (Mueller et al., 2017). Shortly after birth, feeding continues to expose the newborn’s gut to bacteria. And just as differences in diet alter a newborn’s perception of nutrients, the microbiome of breast and formula fed infants may also be affected. Breast feeding has been associated with an increased abundance of bifidiobacteria species, decreased microbial diversity, and increased stability of the microbiota population (Bezirtzoglou et al., 2011; Kashtanova et al., 2016). Alterations of the gut microbiota in this sensitive time have also been shown to contribute to disease; antibiotics administered in early infancy have also been associated with an increased risk for obesity (Bailey et al., 2014; Cox et al., 2014).

The study of germ-free animals has allowed researchers to investigate the role of the microbiota in gut-brain signaling and development (Luczynski et al., 2016). Gnotobiotic zebrafish have reduced levels of serotonin-positive enteroendocrine cells despite normal levels of intestinal epithelial cells, and numbers can be restored by inoculation with a complete microbiome (Bates et al., 2006). In a mouse model of pre-term infants, inoculation of pre-term mice with the microbiota of pre-term human infants who were adequately gaining weight resulted in improved villus height and crypt depth, increased cell proliferation, increased density of Paneth and goblet cells, and improved tight junctions (Yu et al., 2016). Microbiota are also essential for normal brain development. In one study, germ free mice demonstrate increased motor activity, reduced anxiety-like behavior, and increased striatal levels of synaptophysin (a synaptic vesicle maturation and synaptogenesis marker) and PSD-95 (marker for maturation of excitatory synapses). Early colonization of these germ-free mice with microbiota normalized several behavior patterns and synaptophysin and PSD-95 levels, suggesting early microbiota is crucial for normal brain development and behavior (Diaz Heijtz, 2016). Carlson and colleagues recently demonstrated associations between microbial composition and cognitive functioning, as measured by the Mullen Scales of Early Learning, in developmentally normal infants (Carlson et al., 2018). Gut-brain sensory neural circuitry could link the microbiome to its early effects on the development of the gut, the brain, and disease.

## Conclusion

Recently, gut-brain biology has become an attractive scientific field. Most of the work has focused on correlative observations between gut microbiota and the brain. But emerging literature is revealing the neural circuits linking the gut surface and the brain. The discovery of synapses in enteroendocrine cells has provided a new avenue of exploration in how nutrients and microbiota signals in the gut modulate brain function and behaviors. It has also allowed us to look at the development of gut-brain neural circuits from a new perspective. There are significant parallels between enteroendocrine cell and sensory neuron development and differentiation. At an early gestational age, we begin to perceive the outside world through innervated sensory epithelial cells. Enteroendocrine cells are included in this family now. What we feel, hear, taste, smell and eat *in utero* influences our development and later integration into the world at birth. Alterations in the development of these sensory neuroepithelial circuits are potential targets for gut and brain disorders linked to visceral hypersensitivity. Some of those include obesity, anorexia, autism, chronic abdominal pain and irritable bowel disease. After all, when the gut first meets the brain may be when our brain learns to feel food in the gut.

## Author Contributions

KB and DB designed the scope of the review. KB wrote the first draft of the manuscript. DB edited the manuscript.

## Conflict of Interest Statement

The authors declare that the research was conducted in the absence of any commercial or financial relationships that could be construed as a potential conflict of interest.
